# Fate mapping reveals that microglia and recruited monocyte-derived macrophages are definitively distinguishable by phenotype in the retina

**DOI:** 10.1038/srep20636

**Published:** 2016-02-09

**Authors:** E. G. O’Koren, R. Mathew, D. R. Saban

**Affiliations:** 1 Department of Ophthalmology, Duke University School of Medicine, Durham, NC, USA; 2 Department of Immunology, Duke University School of Medicine, Durham, NC, USA

## Abstract

The recent paradigm shift that microglia are yolk sac-derived, not hematopoietic-derived, is reshaping our knowledge about the isolated role of microglia in CNS diseases, including degenerative conditions of the retina. However, unraveling microglial-specific functions has been hindered by phenotypic overlap of microglia with monocyte-derived macrophages. The latter are differentiated from recruited monocytes in neuroinflammation, including retina. Here we demonstrate the use of fate mapping wherein microglia and monocyte-derived cells are endogenously labeled with different fluorescent reporters. Combining this method with 12-color flow cytometry, we show that these two populations are definitively distinguishable by phenotype in retina. We prove that retinal microglia have a unique CD45^lo^ CD11c^lo^ F4/80^lo^ I-A/I-E^−^ signature, conserved in the steady state and during retinal injury. The latter was observed in the widely used light-induced retinal degeneration model and corroborated in other models, including whole-body irradiation/bone-marrow transplantation. The literature contains conflicting observations about whether microglia, including in the retina, increase expression of these markers in neuroinflammation. We show that monocyte-derived macrophages have elevated expression of these surface markers, not microglia. Our resolution of such phenotypic differences may serve as a robust way to help characterize isolated roles of these cells in retinal neuroinflammation and possibly elsewhere in CNS.

The breakthrough discovery of the embryonic origin of microglia in adult mice is redefining our understanding of these cells in immunopathogeneses of neuroinflammation and CNS conditions, including degenerative diseases of the retina. Ginhoux *et al.* formally demonstrated that microglia arise from yolk sac–primitive macrophages and persist into adulthood^1^. Adding to this finding were the important works of Ajami *et al.*^2^ and Mildner *et al.*^3^, who both showed that circulating monocytes (Mo) do not contribute to the microglial pool under normal physiologic conditions, and that local self-renewal sustains microglia maintenance throughout adult life. Ajami *et al.* also showed that bone marrow-derived cells do not contribute to the pool of microglia even following Mo recruitment in inflammation^2^,^3^,^4^ (a noted exception to this observation can occur in the setting of lethal irradiation/bone marrow transplantation^2^,^3^). Considerable attention has been shifted toward redefining the isolated role of microglia, with the understanding that these cells are distinct from recruited Mo-derived cells in inflammatory diseases of the CNS. Early reports point to the possibility of non-redundant roles for these cells^5^, as implicated in models of Alzheimer’s disease^6^, experimental autoimmune encephalomyelitis (EAE)^7^, and in models of retinal degenerative disease^8^,^9^.

Nonetheless, discovering the isolated role of microglia in the brain and the retina has proven to be technically challenging. For one, microglia and certain Mo-derived cells have been deemed phenotypically indistinguishable. Indeed, recruited classical Mo can be resolved phenotypically because they express Ly6C and CCR2, but not F4/80 or Iba-1. However, differentiated Mo-derived macrophages (mo-MFs) overlap phenotypically with microglia because both express F4/80 and Iba-1, but not Ly6C and CCR2^10^. Although microglia expression of CD45 is uniquely low in the steady state^10^, the literature contains conflicting observations about whether microglia, including in the retina, increase expression of markers such as CD45, F4/80, I-A/IE, or CD11c in neuroinflammation^11^,^12^,^13^. Using genetic reporter mice such as *CX*_*3*_*CR1*^*GFP/wt*^ mice to identify CX_3_CR1-expressing microglia can be misleading, because recruited mo-MFs also express CX_3_CR1^14^,^15^,^16^, as shown in the CNS^17^,^18^, including the retina^19^,^20^,^21^,^22^. Such is also the case with using *CCR2*^*RFP/wt*^ mice to identify Mo and Mo-derived cells^23^, as fully differentiated mo-MFs down regulate their expression of CCR2 to negligible levels that are similar to microglia^10^.

Generating GFP bone marrow chimeras is a commonly used non-genetic approach used to discern microglia from recruited Mo-derived cells during injury or disease. However, this method is also confounded since whole-body irradiation/bone marrow transplantation itself leads to recruitment of bone marrow-derived cells into the CNS^2^, including the retina^21^,^22^. Parabiosis circumvents this problem^2^ but does not reach full chimerism in the periphery and is not widely used for multiple reasons^17^, including significant technical challenges. Using pharmacologic (or genetic) blockade of CCR2 as a means to discern microglia-specific functions has been very useful in CNS models^3^, including the retina^9^. However, it spares non-classical Mo that do not express CCR2^24^. Also this approach ablates recruitment at the level of the classical monocyte^15^, and thus the isolated role of mo-MFs cannot be studied. In short, while these systems have been helpful, a better understanding of microglia and recruited mo-MFs is required to overcome the aforementioned technical hurdles and help fill major gaps in our current knowledge regarding fate and function of these cells in neuroinflammation.

In the current study, we utilized a fate mapping technique by crossing a tamoxifen-inducible Cre mouse line (*CX*_*3*_*CR1*^*YFP−CreER/YFP−CreER*^) to a red fluorescence protein (RFP) Cre reporter mouse line (*R26*^*RFP*^)^25^,^26^. We show here that the progeny of this crossing, *CX*_*3*_*CR1*^*YFP−CreER/wt*^*:R26*^*RFP*^, can be used to differentially label microglia vs. recruited Mo-derived cells in the retina. This system works by tamoxifen pulsing mice to temporarily activate Cre in constitutively CX_3_CR1 positive (YFP^+^) cells, i.e. Mo and microglia. The activated Cre removes a stop codon controlling RFP expression such that YFP^+^ cells become RFP^+^. Once the tamoxifen is not longer bioavailable, Cre is inactive and no new YFP^+^ cells can express RFP. This step is followed by a “wash out” period of several weeks wherein RFP expression is lost in Mo (as they turnover in circulation), whereas expression is naturally and indefinitely retained by microglia (because they are long-lived and self-renewing).

By use of this fate mapping system, in conjunction with 12-color flow cytometry, we demonstrate here that microglia in the retina have a distinct CD45^lo^ CD11c^lo^ F4/80^lo^ I-A/I-E^−^ profile. Surprisingly, this profile was conserved during light-induced retinal injury or lethal whole-body irradiation, whereas it was recruited mo-MFs that expressed high levels of such markers in the same settings. Thus, our results establish the use of fate mapping to definitively distinguish retinal microglia vs. recruited mo-MFs and reveal a unique CD45^lo^ CD11c^lo^ F4/80^lo^ I-A/I-E^−^ signature for microglia conserved in the steady state and in retinal neuroinflammation. These findings are likely relevant in other retinal disease models with myeloid cell involvement, such as in autoimmune uveitis, glaucoma, photoreceptor degeneration, age-related macular degeneration, and diabetic retinopathy.

## Results

### Phenotypic characterization of microglia in the normal retina of C57BL/6 mice

Our first aim was to comprehensively phenotype microglia in the normal retina using a multi-parameter flow cytometry panel of currently used myeloid cell markers. Markers included live/dead, CD45 (leukocyte), CD11c (dendritic cell; DC), I-A/I-E (antigen presenting cell), and F4/80 (macrophage; MF). We also incorporated CD64 (MF), also known as FcγRI, which was recently validated as a faithful marker for MFs^27^. In addition, markers such as CD11b (myeloid), Ly6C (Mo), and Ly6G (neutrophil; PMN) were also incorporated to discern Mo and PMN. In subsequent experiments, endogenous reporters (e.g. YFP, GFP, RFP) are also included. In all, a maximum of 12 parameters could be simultaneously analyzed using flow cytometry. In the current experiment, we analyzed normal retina from C57BL/6 adult mice to identify microglia, the predominant myeloid cell population in steady-state CNS parenchyma.

**Figure 1:**
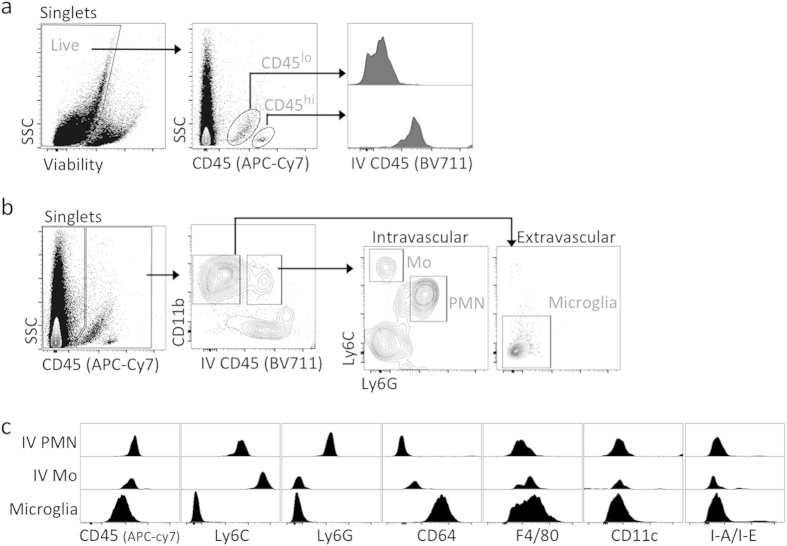
Phenotypic characterization of microglia in the normal retina of C57BL/6 mice. Intravenous anti-CD45 mAb method was used for flow cytometric exclusion of intravascular immune cells within retina^29^. Administration via IV route of CD45 mAb, i.e. IV-CD45 (BV711), was given to C57BL/6 mice 5 minutes before euthanasia, labeling intravascular immune cells of retina. Subsequently harvested retinas were *ex vivo* stained with the remaining conjugated mAbs, including APC-Cy7 conjugated anti-CD45. (**a**) Intravascular IV-CD45^+^ events correspond with CD45^hi^ (APC-Cy7) cells, whereas extravascular IV-CD45^−^ events correspond with CD45^lo^ (APC-Cy7) cells of the retina. Cells were pre-gated on singlets. (**b**) IV-CD45^+^ cells contain circulating myeloid cells such as classical monocytes (Mo) and neutrophils (PMN), whereas IV-CD45^−^ cells contain microglia. Cells were pre-gated on live singlets. (**c**) Evidence for a CD45^lo^ CD11c^lo^ F4/80^lo^ I-A/I-E^−^ profile for retinal microglia. Extravascular IV-CD45^−^ CD11b^+^ cells were compared to intravascular IV-CD45^+^ CD11b^+^ Mo and PMN, and examined for expression of the indicated markers. Flow cytometry plots are comprised of concatenated data from n = 4 retinas to increase the number of events for intravascular cell gating. Data is representative of at least 2 independent experiments, each with n = 4 individual retina samples.

To discriminate extravascular myeloid cells (i.e. microglia) from intravascular ones (i.e. Mo and PMN) by flow cytometry, we used an approach previously described by Jakubzick *et al.*^28^ and later validated by our group with ocular posterior segment tissues^29^. This method involves intravenous (IV) infusion with anti-CD45 mAb (biotinylated) several minutes prior to euthanasia and subsequent *ex vivo* staining with BV711-conjugated streptavidin; labeling is referred to as IV-CD45. *Ex vivo* staining with other aforementioned conjugated mAbs was also performed concurrently, including APC Cy7-conjugated anti-CD45. Data showed that IV-CD45^+^ cells indeed corresponded with CD45^hi^ myeloid populations found in circulation, including classical Mo (CD11b^+^ Ly6G^−^ Ly6C^+^) and PMN (CD11b^+^ Ly6G^+^ Ly6C^lo^) (Fig. 1a,b). In contrast, IV-CD45^−^ cells corresponded with microglia, as these cells are CD45^lo^ and CD11b^+^ Ly6G^−^ Ly6C^−^ cells (Fig. 1a,b). Using this system, we next directly compared the phenotype of microglia against intravascular Mo and PMN. The complete phenotype of intravascular PMN was IV-CD45^+^ CD11b^+^ Ly6G^+^ Ly6C^lo^ CD64^−^ CD45^hi^ CD11c^lo^ F4/80^−^ I-A/I-E^−^; and intravascular Mo was IV-CD45^+^ CD11b^+^ Ly6G^−^ Ly6C^hi^ CD64^−^ CD45^hi^ CD11c^lo^ F4/80^−^ I-A/I-E^−^ (Fig. 1c). In contrast, microglia were IV-CD45^−^ CD11b^+^ Ly6G^−^ Ly6C^−^ CD64^+^ CD45^lo^ CD11c^lo^ F4/80^lo^ I-A/I-E^−^ (Fig. 1c). Thus, our method allows distinct resolution of microglia from circulating myeloid cells and confirms that microglia have a CD45^lo^ CD11c^lo^ F4/80^lo^ I-A/I-E^−^ profile in the steady-state retina.

### In the setting of whole-body lethal irradiation/bone marrow transplantation, microglia are phenotypically different than recruited mo-MFs

The next aim was to apply our flow cytometric strategy to determine whether recruited Mo-derived cells are phenotypically different from microglia. Our approach was to use whole body lethal-irradiation of host mice followed by donor GFP^+^ bone marrow transplantation (Rad/GFP-BMT). Donor mice ubiquitously expressed GFP driven by the β-actin promoter. This approach offers several advantages. First, a direct effect of this procedure is low-grade chronic injury of the retina^21^ which allows us to gain insight regarding myeloid phenotypes in this injury setting. Second, the procedure itself leads to CCL2-mediated recruitment of Mo into the retina^21^, allowing us to directly compare the phenotypes of GFP^+^ (i.e. donor) Mo-derived cells with GFP^−^ (i.e. host) microglia.

**Figure 2:**
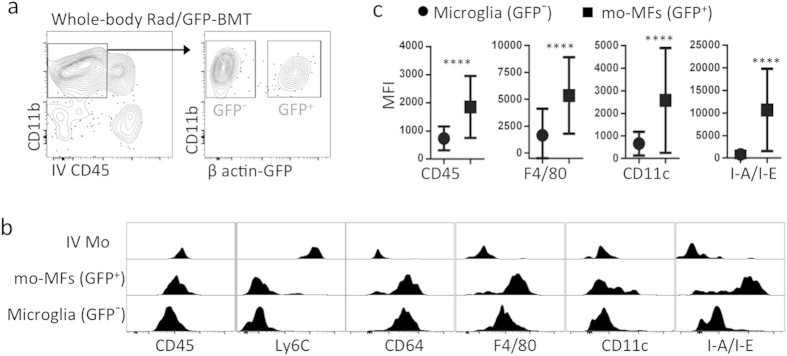
Microglia and recruited mo-MFs are phenotypically distinguishable in the setting of whole-body irradiation and bone marrow transplantation (Rad/BMT). (**a**) Whole-body Rad/BMT leads to recruitment of myeloid cells into the retina. C57BL/6 mice were whole-body irradiated then grafted with GFP^+^ donor bone marrow (Rad/GFP-BMT). Using this method, we can firmly establish GFP^+^ cells as donor-derived whereas all GFP^−^ cells (including microglia) are host-derived. After Rad/GFP-BMT, mice were rested for more than 3 months. Data is representative of 2 independent experiments. Cells were pre-gated on live CD45^+^ singlets. (**b**) Comprehensive phenotypic analysis reveals that microglia have a CD45^lo^ CD11c^lo^ F4/80^lo^ I-A/I-E^−^ profile in the whole-body Rad/GFP-BMT setting. GFP^−^ microglia were compared to extravascular GFP^+^ mo-MFs and intravascular classical Mo (IV Mo). (**c**) MFI indicates that the CD45^lo^ CD11c^lo^ F4/80^lo^ I-A/I-E^−^ profile of GFP^−^ microglia is significantly different from GFP^+^ mo-MFs. Data shown (mean ± s.d.) is a representative sample from n ≥ 4 individual samples/group. Significant differences were determined within samples by an unpaired t test (****p < 0.0001). Data is representative of 2 independent experiments.

C57BL/6 hosts were therefore subjected to whole-body Rad/GFP-BMT, and retinas were collected 3 months later. Data showed a significant population of extravascular myeloid (CD11b^+^) cells in the retina that were GFP^+^ (i.e. donor) at this time-point and a large population of remaining GFP^−^ (i.e. host) microglia (Fig. 2a). With respect to intravascular GFP^+^ classical Mo, their phenotype was IV-CD45^+^ CD11b^+^ Ly6G^−^ Ly6C^hi^ CD64^−^ CD45^+^ CD11c^lo^ F4/80^−^ I-A/I-E^−^ (Fig. 2b). We were not able to detect an appreciable presence of extravasated GFP^+^ classical Mo (data not shown), however, GFP^+^ mo-MFs were readily detectable and their phenotype was IV-CD45^−^ CD11b^+^ Ly6G^−^ Ly6C^−^ CD64^+^ CD45^+^ CD11c^+^ F4/80^+^ I-A/I-E^+^ (Fig. 2b). In contrast, GFP^−^ microglia were IV-CD45^−^ CD11b^+^ Ly6G^−^ Ly6C^−^ CD64^+^ CD45^lo^ CD11c^lo^ F4/80^lo^ I-A/I-E^−^ (Fig. 2b). These data revealed unexpected potential differences in expression levels of CD45, CD11c, F4/80 and I-A/I-E in microglia vs. mo-MFs, which was verified by comparing their mean fluorescence intensities (MFI) (Fig. 2c). MFI data of each individual sample was determined (Fig. S1). Thus, our data suggests that, in the setting of whole-body Rad/GFP-BMT, microglia have a unique CD45^lo^ CD11c^lo^ F4/80^lo^ I-A/I-E^−^ profile, and that their profile is not shared by recruited mo-MFs, which express significantly higher levels of these markers.

### In light injury, microglia are phenotypically different from mo-MFs, as revealed in shielded Rad/GFP-BMT hosts

**Figure 3:**
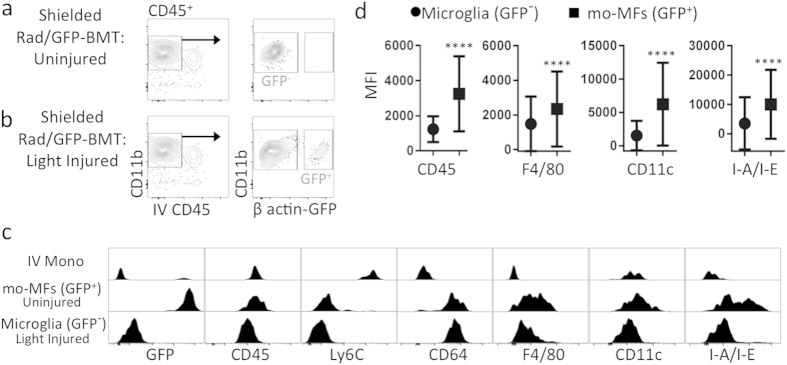
Microglia and recruited mo-MFs are phenotypically distinguishable in light injury, as revealed in shielded Rad/GFP-BMT hosts. (**a**) Head shielding protects host mice from radiation and radiation-induced Mo recruitment. C57BL/6 mice were irradiated with shielding then given donor GFP^+^ bone marrow. Retinas were analyzed 3 months later. Cells were pre-gated on live CD45^+^ singlets. (**b**) Light injury leads to myeloid cell recruitment in shielded Rad/GFP-BMT hosts. Three months after shielded Rad/GFP-BMT, mice were then subjected to light challenge (or not) and retinas were harvested 7 days later for analysis. Cells were pre-gated on live CD45^+^ singlets. (**c,d**) Comprehensive phenotypic analysis reveals that microglia have a CD45^lo^ CD11c^lo^ F4/80^lo^ I-A/I-E^−^ profile during light injury, which is significantly different from recruited mo-MFs. GFP^−^ microglia from retina of uninjured mice were compared to GFP^+^ mo-MFs and IV Mo from retinas of light-injured mice. Significant differences of data shown (mean ± s.d.) were determined across samples by an unpaired t test (****p < 0.0001). Light injury data is representative of n = 4 individual samples; pre-light injury data is comprised of n = 2 individual samples that are representative of 2 independent experiments.

Our next aim was to examine the phenotype of microglia and recruited mo-MFs in a more acute and robust injury setting. However, we first had to modify our whole-body Rad/GFP-BMT fate mapping technique because, as shown above, the whole-body approach causes retinal injury^21^ and recruitment of mo-MFs. Therefore, we shielded the heads of host mice during lethal irradiation, as previously described^3^. We then validated that at 3 months post shielded Rad/GFP-BMT, host mice were protected from irradiation-induced recruitment of GFP^+^ donor cells into the retina (Fig. 3a). As an alternative method of injury, we utilized a common light injury model wherein mice are exposed to toxic levels of bright white light that cause acute photoreceptor cell death^30^. The advantages of this model include that injury occurs primarily at the level of the neuroretina (specifically the photoreceptors)^30^ and that myeloid cells are significantly involved^9^,^31^,^32^. Thus, the following experiment applied light injury in shielded Rad/GFP-BMT hosts to enable us to discern newly recruited Mo-derived cells from microglia in the retina. The LED light injury model utilized here is described in greater detail in the methods sections.

Shown here, shielded Rad/GFP-BMT was performed and then 3 months later host mice were exposed to a light challenge (or left unchallenged) and retinas were collected 7 days later, a time-point consistent with substantial light-induced injury (data not shown). Agreeing with our previous experiments, we observed a significant population of extravascular donor-derived GFP^+^ myeloid cells in light injury and a large population of host-derived GFP^−^ microglia (Fig. 3b). For more in depth phenotypic examination, we compared mo-MFs and microglia from retinas of shielded Rad/GFP-BMT hosts, in uninjured vs. light-injured mice; specifically, we compared recruited GFP^+^ myeloid cells from light-injured retinas vs. GFP^−^ microglia from uninjured counterparts (Fig. 3a). The latter was necessary to avoid contamination of GFP^−^ recruits in our analysis, as full chimerism in shielded Rad/GFP-BMT cannot be achieved (Fig. S2a). With respect to intravascular Mo, their phenotype was IV-CD45^+^ CD11b^+^ Ly6G^−^ Ly6C^hi^ CD64^−^ CD45^+^ CD11c^lo^ F4/80^−^ I-A/I-E^−^ (Fig. 3c). Recruited mo-MFs were readily detectable and their phenotype was IV-CD45^−^ CD11b^+^ Ly6G^−^ Ly6C^−^ CD64^+^ CD45^+^ CD11c^+^ F4/80^+^ I-A/I-E^+^ (Fig. 3c). In contrast, microglia were IV-CD45^−^ CD11b^+^ Ly6G^−^ Ly6C^−^ CD64^+^ CD45^lo^ CD11c^lo^ F4/80^lo^ I-A/I-E^−^ (Fig. 3c). Once again, we verified via MFI analysis that the expression levels of CD45, CD11c, F4/80 and I-A/I-E in microglia vs. mo-MFs were significantly different (Fig. 3d). MFI data of each sample was determined (Fig. S2b). Thus, as revealed by shielded Rad/GFP-BMT hosts, our data showed that in the light injury setting, microglia have a unique CD45^lo^ CD11c^lo^ F4/80^lo^ I-A/I-E^−^ profile. Furthermore this phenotype was not shared by recruited mo-MFs, which express significantly higher levels of these markers.

### Fate mapping with CX_3_CR1^YFP*−*CreER/wt^:R26^RFP^ hosts corroborates that microglia are phenotypically different from mo-MFs in light injury

**Figure 4:**
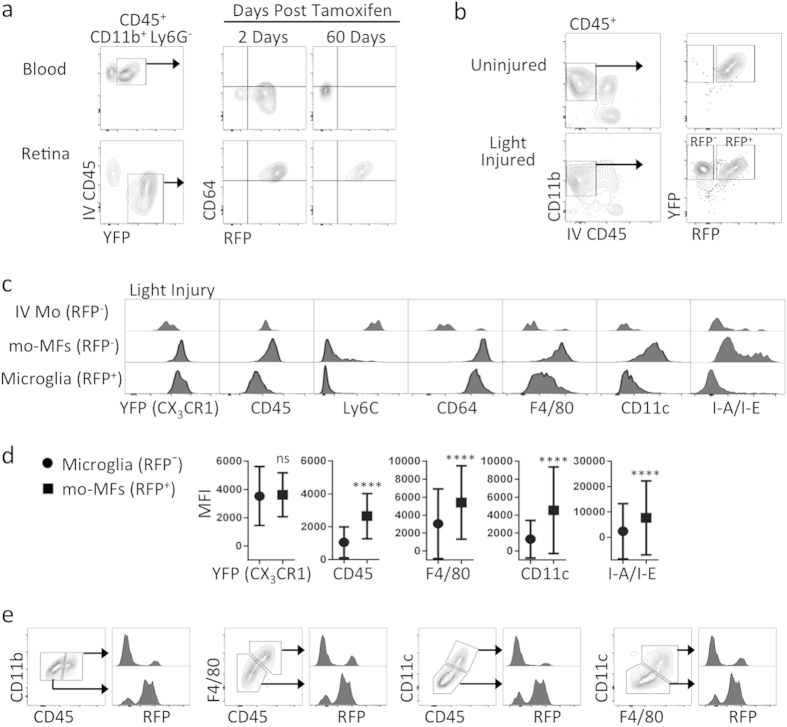
Fate mapping with *CX*_*3*_*CR1*^*YFP−CreER/wt*^*:R26*^*RFP*^ mice reveals that microglia and recruited mo-MFs are phenotypically distinguishable in light injury. (**a**) Fate mapping in *CX*_*3*_*CR1*^*YFP−CreER/wt*^*:R26*^*RFP*^ mice differentially label microglia (i.e. YFP^+^ RFP^+^) vs. blood Mo (i.e. YFP^+^ RFP^−^), which is achieved by tamoxifen administration followed by a “wash out” period. Tissues were harvested 2 days after the last dose, or following a 60 day “wash out” period. Cells were pre-gated on live CD45^+^ CD11b^+^ Ly6G^−^ singlets. (**b**) Fate mapping discerns microglia from recruited Mo-derived cells in light injured retinas. Following tamoxifen pulsing and subsequent “wash out” period, mice were subjected to light challenge (or not) and retinas were harvested 5 days later. (**c,d**) RFP^+^ microglia were compared to RFP^−^ mo-MFs and IV Mo. Comprehensive phenotypic analysis reveals that microglia have a CD45^lo^ CD11c^lo^ F4/80^lo^ I-A/I-E^−^ profile during light injury, which is significantly different from recruited mo-MFs. Data from uninjured and light-injured retina are representative of n = 2 and n = 5 individual samples, respectively. Data is representative of two independent experiments. Significant differences were determined within samples by an unpaired t test (****p < 0.0001). (**e**) Microglia and mo-MFs are distinguishable by surface staining of CD45, F4/80, or CD11c. Gated on extravascular CD11b^+^ retinal cells. Data shows different gating strategies using CD11b, CD45, and F4/80 to identify high and low expression cell populations. Subsequently, we analyzed RFP expression in each population to determine if the gating corresponds to genetic fate mapping. Cells were pre-gated on live CD45^+^ CD11b^+^ singlets. Data is representative of at least 2 independent experiments; each experiment has n = 4 individual samples.

Our next aim was to use an alternative approach for tracking myeloid cell origins to possibly corroborate that microglia have a unique CD45^lo^ CD11c^lo^ F4/80^lo^ I-A/I-E^−^ profile not shared with mo-MFs. We generated *CX*_*3*_*CR1*^*YFP−CreER/wt*^*:R26*^*RFP*^ mice to utilize a previously described fate mapping strategy accomplished by pulsing mice with tamoxifen to label CX_3_CR1 expressing cells (i.e. YFP^+^ cells) with RFP. Indeed, we found that 2 days after tamoxifen administration, YFP^+^ circulating Mo and YFP^+^ retinal microglia both expressed RFP (Fig. 4a). However, 60 days post tamoxifen administration, only retinal microglia maintained RFP expression (Fig. 4a); whereas RFP expression in circulating Mo was “washed out” by 60 days (Fig. 4a). Thus, at the 60-day time-point, retinal microglia and circulation Mo exhibited a YFP^+^ RFP^+^ and YFP^+^ RFP^−^ phenotype, respectively. Next, we exposed “washed out” mice to light challenge and analyzed retinas 5 days later. We observed a YFP^+^ RFP^+^ population consistent with microglia and a YFP^+^ RFP^−^ population consistent with recruited Mo-derived cells (Fig. 4b). In contrast, mice that were not exposed to light challenge only possessed YFP^+^ RFP^+^ microglia (Fig. 4b). Using flow cytometric enumeration, we observed that the total number of YFP^+^ cells in the retina significantly doubled after injury (Fig. S3a). Interestingly, when we enumerated YFP^+^ RFP^+^ microglia vs. YFP^+^ RFP^−^ mo-MFs, there was no significant increase in microglia, suggesting that the observed increase in overall cellularity was entirely a result of newly recruited cells (Fig. S3a).

Next, we performed more in depth examinations of retinal cell phenotypes after light injury. With respect to intravascular Mo (YFP^+^ RFP^−^), their phenotype was IV-CD45^+^ CD11b^+^ Ly6G^−^ Ly6C^hi^ CD64^−^ CD45^+^ CD11c^lo^ F4/80^−^ I-A/I-E^−^ (Fig. 4c). We also identified a small population of extravascular YFP^+^ RFP^−^ Ly6C^+^ Mo-derived cells (Fig. S3b). However, these cells expressed F4/80 and CD64 (Fig. S3c), suggesting that their differentiation into mo-MFs was already underway. RFP^−^ Ly6C^−^ mo-MFs were readily detectible and their phenotype was IV-CD45^−^ CD11b^+^ Ly6G^−^ Ly6C^−^ CD64^+^ CD45^+^ CD11c^+^ F4/80^+^ I-A/I-E^+^ (Fig. 4c). In contrast, RFP^+^ microglia were IV-CD45^−^ CD11b^+^ Ly6G^−^ Ly6C^−^ CD64^+^ CD45^lo^ CD11c^lo^ F4/80^lo^ I-A/I-E^−^ (Fig. 4c). Again, we were able to verify via MFI analysis that the expression levels of CD45, CD11c, F4/80 and I-A/I-E in microglia vs. mo-MFs were significantly different (Fig. 4d). MFI data of each individual sample was determined (Fig. S3d). Moreover, we were able to demonstrate that these populations can be discerned without fate mapping (Fig. 4e). Data shows gating of high and low expressing populations using CD45 alone, CD45 in combination with F4/80 or CD11c, and F4/80 in combination with CD11c. Based on these gates, we assessed the expression of RFP. Consistently, the gated population with lower CD45, F4/80, or CD11c contained the vast majority of RFP^+^ microglia with minimal spillover of RFP- mo-MFs. Conversely, the gated population with higher CD45, F4/80, or CD11c contained the vast majority of RFP^−^ mo-MFs with minimal spillover of microglia. Finally, we directly compared microglia from the steady state to those from light-injured retina, addressing whether any shifts in expression were detectable. Results indicate that changes in expression, if any, were marginal as compared to mo-MFs (Fig. S4a,b). Fluorescence minus one controls were included (Fig S4a,b).

**Figure 5:**
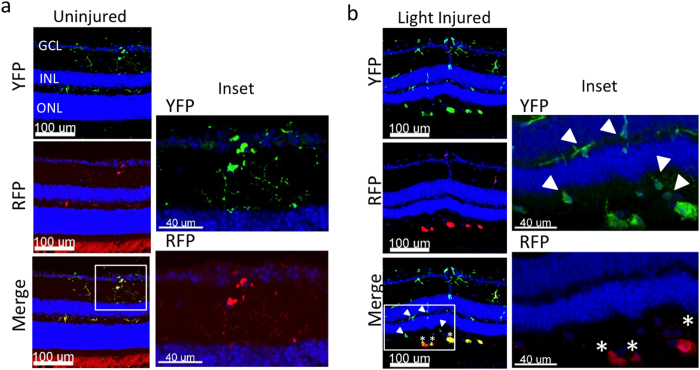
Fate mapping with *CX*_*3*_*CR1*^*YFP−CreER/wt*^*:R26*^*RFP*^ mice using confocal analysis. Following tamoxifen pulsing and a “wash out” period, mice were subjected to light challenge (or not) and retinas were harvested 5 days later. (**a**) Uninjured retinas contain microglia (inset: YFP^+^ RFP^+^) and display normal retinal architecture, as visualized by DAPI (outer nuclear layer = ONL; inner nuclear layer = INL; ganglion cell layer = GCL). (**b**) Light injured retinas have a thin ONL and possess microglia and recruited mo-MFs (inset: YFP^+^ RFP^+^ and YFP^+^ RFP^−^, respectively). Asterisks indicate microglia; arrowheads indicate recruited mo-MFs. Data is representative of two independent experiments; each experiment contained data from individual samples, uninjured n = 2 and injured n = 5.

Next, the ability to discern YFP^+^ RFP^+^ microglia and recruited YFP^+^ RFP^−^ Mo-derived cells in *CX*_*3*_*CR1*^*YFP−CreER/wt*^*:R26*^*RFP*^ mice was also verified by confocal analysis (Fig. 5). Taken together, we revealed that in the light injury setting, microglia have a unique CD45^lo^ CD11c^lo^ F4/80^lo^ I-A/I-E^−^ profile which is not shared by recruited mo-MFs.

Lastly, we showed that our flow cytometric strategy is applicable to other mouse strains. Exposure to 60 k lux for 8 hr was required to damage the retina in mice on the C57Bl/6 background, e.g. *CX*_*3*_*CR1*^*YFP−CreER/wt*^*:R26*^*RFP*^ mice (Fig. 5b). In contrast, mouse strains known to be susceptible to light injury, like BALB/c and CB6F1J^33^,^34^, required only 40 k lux for 4 hr (Fig. S6b). Similar to our results in *CX*_*3*_*CR1*^*YFP−CreER/wt*^*:R26*^*RFP*^, damaged CB6F1J mice showed invasion of myeloid cells to the outer retina (Fig. S6c). We performed more in depth examinations of retinal cell phenotypes after 5 days post light injury in CB6F1/J mice. Data show gating of high and low expressing populations using CD45 in combination with CD11c (Fig. S6d). The CD45^hi^ CD11c^+^ population was not present in baseline retina, suggesting that it was a newly recruited population. We lacked fate mapping abilities in the CB6F1/J mice, but we analyzed the expression of F4/80 and I-A/I-E on the gated populations (Fig. S6e). Consistent with the phenotype of recruited Mo-derived cells, the CD45^hi^ CD11c^hi^ population was also F4/80^+^ I-A/I-E^+^. And consistent with the phenotype of microglia, the CD45^lo^ CD11c^lo^ population was F4/80^lo^ I-A/I-E^−^. Taken together our data suggest that microglia and Mo-derived cells have discernible phenotypes across mouse strains.

### Analysis of extravascular I-A/I-E^hi^ myeloid cells in normal retina

Over the course of our experiments, we noticed a small but consistent subpopulation of extravascular I-A/I-E^hi^ myeloid cells in the normal retina. We devoted the last series of experiments to further analyzing this subpopulation. A previous report by Lehmann *et al.* described a population of retinal cells in *CD11c-DTR/GFP* mice as having GFP and MHC II expression^35^. Thinking these cells might correspond to the I-A/I-E^hi^ myeloid cells that we observed, we also surveyed retinas of *CD11c-DTR/GFP* mice. Our data clearly showed a small but distinct population of GFP^+^ myeloid cells (Fig. S5a,b), which were specifically deleted following systemic diphtheria toxin (DT) administration (Fig. S1a,b). Interestingly, the phenotype of these GFP^+^ cells was identical to GFP^−^ cells; IV-CD45^−^ CD11b^+^ Ly6G^−^ Ly6C^−^ CD64^+^ CD45^lo^ CD11c^lo^ F4/80^lo^ I-A/I-E^−^ (Fig. S1c) Furthermore, the I-A/I-E^hi^ subpopulation was detectable in both the GFP^+^ and GFP^−^ fractions, and thus was not uniquely associated with either population.

**Figure 6:**
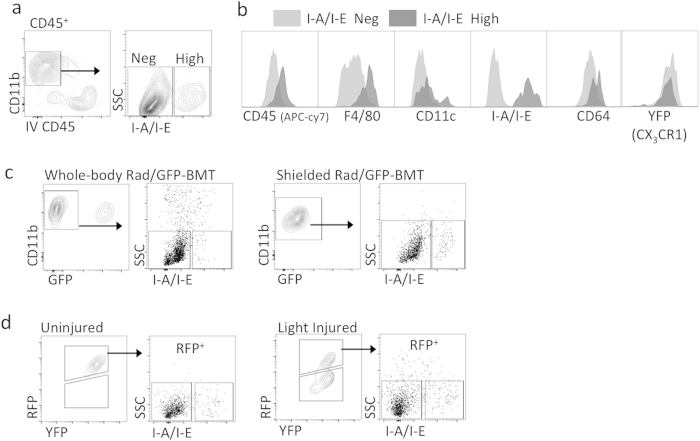
Analysis of extravascular I-A/I-E^hi^ myeloid cells in normal retina. (**a**) Identification of I-A/I-E^hi^ myeloid cells in the retina. Cells were pre-gated on live CD45^+^ singlets. (**b**) I-A/I-E^hi^ cells in the normal retina are putative MFs, as indicated by positive CD64 and F4/80 expression. Data is representative of two independent experiments; each experiment has n = 4 individual samples. (**c**) Evaluation of I-A/I-E^hi^ putative MFs, regarding long-lived and radio-resistant status. Mice were subjected to whole-body or shielded Rad/GFP-BMT then retinas were harvested 3 months later. I-A/I-E^hi^ putative MFs were evident following shielded, but not whole-body, Rad/GFP-BMT. Cells were pre-gated on live CD45^+^ singlets. Data is representative of 2 independent experiments; each plot is representative of 2 to 4 individual samples. (**d**) Fate mapping in *CX*_*3*_*CR1*^*YFP−CreER/wt*^*:R26*^*RFP*^ mice to further evaluate possible long-lived status of I-A/I-E^hi^ putative MFs. Mice were tamoxifen pulsed. Then after 3 months, mice were subjected to light challenge (or not) and retinas were harvested 5 days later for flow cytometry analyses. Long-lived RFP^+^ cells were analyzed for the presence/absence of I-A/I-E^hi^ putative MFs. Cells were pre-gated on live CD45^+^ CD11b^+^ singlets. Data is representative of two independent experiments; each experiment contained data from individual samples, uninjured n = 2 and injured n = 5.

Next, we performed an in depth phenotypic analysis of the I-A/I-E^hi^ subpopulation by directly comparing these cells with I-A/I-E^−^ cells (microglia) (Fig. 6a). Extravascular myeloid cells from the retina of C57BL/6 mice were separately gated based on I-A/I-E^hi^ or I-A/I-E^−^ expression (Fig. 6a). Subsequent phenotypic analysis showed that the expression of several markers was increased in the I-A/I-E^hi^ population (Fig. 6b), and thus appeared to be phenotypically distinct from I-A/I-E^−^ microglia. We also noted the I-A/I-E^hi^ population expressed CD64 and F4/80 (Fig. 6b), thus allowing us to designate these cells as putative MFs^27^.

To assess whether the I-A/I-E^hi^ putative MFs might be long-lived and radio-resistant, like microglia, we subjected mice to either whole-body or shielded Rad/GFP-BMT. Three months later, the retinas of these mice were analyzed. We gated on IV-CD45^−^ CD11b^+^ GFP^−^ events (Fig. 6c), excluding GFP^+^ myeloid recruits from our phenotypic analysis. Results showed that the presence of the I-A/I-E^hi^ putative MFs following shielded Rad/GFP-BMT hosts was abundantly clear (Fig. 6c), suggesting that these cells are long-lived and consistent with the MF phenotype. In contrast, the presence of these putative MFs following whole-body Rad/GFP-BMT was rather marginal (Fig. 6b), suggesting that these cells are not radio-resistant.

To further probe the possibility of a long-lived status for such I-A/I-E^hi^ putative MFs, we employed our fate mapping technique with *CX*_*3*_*CR1*^*YFP−CreER/wt*^*:R26*^*RFP*^ mice. Mice were tamoxifen pulsed and analyzed 3 months later after “wash out”. We also included a cohort of mice that were light injured. After gating on RFP^+^ (i.e. long-lived) cells, a distinct population of I-A/I-E^hi^ cells was observed both in normal and in light-injured retina (Fig. 6d). Taken together, these data suggest that I-A/I-E^hi^ putative MFs are long-lived, albeit not radio-resistant, whereas I-A/I-E^−^ microglia are long-lived and radio-resistant.

## Discussion

The current study addresses unresolved questions concerning the presence of *bona fide* microglia vs. recruited mo-MFs and their possible phenotypic differences in the injured retina. Our novel work involves the use of fate mapping with *CX*_*3*_*CR1*^*YFP−CreER/wt*^*:R26*^*RFP*^ mice or bone marrow chimeras, in conjunction with multi-parameter flow cytometric analyses to address these questions. We established a technique that uses IV infusion of CD45 mAb to tag circulating leukocytes (i.e. Mo, PMN, etc.) within retinal vasculature, which facilitates subsequent exclusion of these cells by flow cytometry analysis. In combining such tools, our approach enabled us to directly demonstrate in retinal injury that microglia (i.e. embryonically-derived/tissue-resident) are distinct from recruited mo-MFs (adult bone marrow-derived/recruited). These two populations have only recently been accepted as ontogenically separate cell populations and are currently considered by many to be phenotypically indistinguishable. We show in the normal retina that microglia have a unique CD45^lo^ CD11c^lo^ F4/80^lo^ I-A/I-E^lo^ profile that is maintained in two different types of injury, chronic/low-grade retinal injury^21^ (whole-body Rad/BMT model) and acute/robust retinal injury (light challenge model^30^). Thus, the CD45^lo^ CD11c^lo^ F4/80^lo^ I-A/I-E^lo^ signature distinguishes microglia from recruited mo-MFs with high fidelity and this phenotype may serve to be a helpful way in future studies to discern these two populations in retina and possibly elsewhere in the CNS by flow cytometry.

Our results regarding microglial phenotype in retinal injury from whole-body Rad/BMT or light-induced damage, challenge the dogma that these cells upregulate certain markers in the neuroinflammed setting. The complete phenotype of microglia in the retina demonstrated here, is as follows: IV-CD45^−^ CD11b^+^ Ly6G^−^ Ly6C^−^ CX_3_CR1^+^ CD64^+^ CD45^lo^ CD11c^lo^ F4/80^lo^ I-A/I-E^−^. This expression profile agrees with that of normal brain microglia, as demonstrated by Gautier *et al.*^27^. Strikingly, however, we found that their CD45^lo^ CD11c^lo^ F4/80^lo^ I-A/I-E^−^ expression is maintained following injury, even when we directly compared microglia from steady-state vs. injured retina. Thus, the previously held notion that activated microglia increase their expression of these markers in the photoreceptor degeneration setting^12^,^36^,^37^,^38^ is in contrast with our light damage data. Also, we made the same finding in the whole-body Rad/BMT setting, which may suggest the presence of a generalized phenomenon. For example, microglia are thought to increase MHC II expression (referred to here as I-A/I-E) in models of EAE^39^,^40^,^41^, and in models of glaucoma and retinal CMV infection^42^,^43^. Likewise, microglia in EAE are thought to upregulate CD45^39^,^44^ and CD11c^39^. The latter is also believed to increase on microglia in models of cuprizone injury^45^, and Alzheimer’s^46^. However, our data suggest that myeloid cells with increased expression of such markers may instead be monocyte-derived. Our combined use of multi-color flow cytometry (including our IV-CD45 technique) with fate mapping strategies provided optimal resolution to discern microglia from extravasated mo-MFs. Our finding has functional implications as it is well documented that surface level expression of receptors impact responses (e.g. I-A/I-E levels often correlate with antigen presentation efficiency). Additionally, the fate mapping strategy demonstrated here will be an invaluable tool for future characterization of the isolated role(s) of microglia vs. mo-MFs in retinal diseases, such as certain forms of uveitis, photoreceptor degeneration, age-related macular degeneration, and diabetic retinopathy.

Another very interesting finding made here was the identification of a I-A/I-E^hi^ myeloid population in the normal retina, which our data suggest are long-lived, but not a radio-resistant, MFs. Their long-lived characteristic was revealed by maintenance of RFP expression in *CX*_*3*_*CR1*^*YFP−CreER/wt*^*:R26*^*RFP*^ mice, as well as in shielded Rad/GFP-BMT hosts. A microglial designation for these cells would be in agreement with original flow cytometry studies by Sedgwick and colleagues, who identified a similar I-A/I-E^hi^ population deemed microglia in brain and spinal chord of normal Brown Norway rats^41^. However, in our study, these I-A/I-E^hi^ MFs had substantially elevated levels of F4/80 (and other markers), which we believe differentiates them from microglia. Furthermore, unlike microglia, these cells appeared to be radiosensitive. Supporting the notion that these cells are not microglia is an early report by Dick *et al.*, who demonstrated an I-A/I-E^hi^ population in the normal rat retina that co-expressed CD163 (i.e. ED2)^47^, a marker associated with perivascular MFs based on previous reports^48^. Likewise, confocal microscopy work by Xu *et al.* visualized a population of perivascular I-A/I-E expressing cells in normal mouse retina^49^, although some expressed the lymphoid-resident DC marker DCIR2 (i.e. 33D1). Additionally, work by Lehmann *et al.* demonstrated the presence of perivascular I-A/I-E expressing cells in normal *CD11c-DTR/GFP* retinas and observed that the cells were also GFP^+^^35^, although authors referred to these cells as DCs not MFs. Similarly, we also detected an I-A/I-E^hi^ population of GFP^+^ cells in *CD11c-DTR/GFP* retinas. Thus, it could be that perivascular MFs in steady-state retina are comprised of long-lived, radio-sensitive, I-A/I-E^hi^ cells, which could be relevant in vascular homeostasis and diseases of the retina, such as in diabetic retinopathy.

Regarding recruited mo-MFs in retinal inflammation, our study demonstrates that these cells, not microglia, definitively express increased levels of CD45, CD11c, F4/80 and I-A/I-E following whole-body Rad/BMT or light challenge. Our IV-CD45 technique allowed us to resolve their likely precursors within the retinal vasculature, i.e. circulating classical monocytes (IV-CD45^+^ CD11b^+^ Ly6G^−^ Ly6C^hi^ CX3CR1^+^ CD64^−^ CD45^+^ CD11c^−^ F4/80^−^ I-A/I-E^+^), from recruited mo-MFs (IV-CD45^−^ CD11b^+^ Ly6G^−^ Ly6C^−^ CX_3_CR1^+^ CD64^+^ CD45^+^ CD11c^+^ F4/80^+^ I-A/I-E^+^). Our data also suggest that expression of CX_3_CR1 cannot be used to distinguish microglia and mo-MFs, as both expressed similar levels following light injury. We designated the new recruits as mo-MFs due to their strong F4/80 and CD64 expression. However, they also express CD11c, a marker traditionally associated with DCs but is now recognized to be expressed by certain MFs^27^. The CD11c expression on mo-MFs makes these recruits the likely equivalents to CD11c^+^ recruits previously described in photoreceptor degeneration by others. For example, Chen *et al.* reported the presence of YFP+ cells in the retina of CD11c-YFP mice; a strain of mice that was showed to posses an RD8 mutation known to cause photoreceptor degeneration^50^. Another example includes GFP^+^ cells in light-injured retinas of *CD11c-DTR/GFP* mice^35^. Interestingly, our data may reflect subtle variations in mo-MF phenotypes in response to injury. When contrasting CD11c expression by mo-MFs after light injury vs. whole-body Rad/GFP-BMT, we observed a trend for reduced CD11c expression after whole-body Rad/GFP-BMT, albeit still higher than microglial levels. The latter may be a reflection of chronic/low-grade inflammation evoked by whole-body Rad/GFP-BMT^21^, and indicates that there can be variability in recruited mo-MF phenotypes over time.

Continuing on the theme of recruited cells, our data may suggest the presence of an exceedingly rapid Mo to MF differentiation in the retina. Appreciation for this conceivable phenomenon was prompted from the rarity by which we were able to detect extravasated undifferentiated classical Mo (i.e. Ly6C^hi^ F4/80^−^) following whole-body Rad/GFP-BMT or light challenge. Sparsely present extravascular Ly6C^hi^ cells (also CCR2^+^) were instead found here to be F4/80^+^ and CD64^+^, suggesting that these Mo-derived cells had already begun differentiation. It is tempting to speculate that such a rapid Mo to MF differentiation is a unique feature of inflammation in the retina or CNS as a whole. This concept would be supported in the work by London *et al.*, who detected the presence of mo-MFs in the retina within 12 hr post glutamate intoxication^19^ and Howe *et al.*, who detected these cells in brain parenchyma within 18 hr post intracerebral TMEV inoculation^51^. In contrast to the CNS, in peripheral tissue inflammation, mo-MFs emerge approximately 72 hr after, as seen in models of experimental colitis^14^, myocardial infarction^52^ or corneal wounding in mice^53^. In myocardial infarction and corneal wounding classical monocytes (i.e. Ly6C^hi^ F4/80^−^) are detectable out to 7 days post injury, which was not the case in the retina following light injury in our study. Future work is required to validate this theory of an exceedingly rapid Mo to MF differentiation in the retina and to determine the functional relevance.

In summary, we directly demonstrate through fate mapping and bone marrow chimeras, in conjunction with 12 color flow cytometry, that microglia are distinct from recruited mo-MFs. Our data also identifies a population of long-lived I-A/I-E^hi^ putative MFs in the steady state, which may implicate a perivascular MF designation. In addition, our data also led us to appreciate the possible existence of a profoundly rapid Mo to MF differentiation in the retina. Most importantly, we prove that retinal microglia have a unique CD45^lo^ CD11c^lo^ F4/80^lo^ I-A/I-E^−^ signature that is conserved in both steady state and during injury, which breaks from the traditional dogma that microglia increase expression of these markers in neuroinflammation. Instead, we show that it is the recruited mo-MFs that have elevated expression levels of CD45, CD11c, F4/80, and I-A/I-E. These microglia and mo-MFs have only recently been accepted as distinct and are considered by many to be phenotypically indistinguishable. However, the phenotypic differences shown here may serve as a way to henceforth characterize the isolated role/s of such cells in retinal diseases pathologies, such as uveitis, photoreceptor degeneration, age-related macular degeneration, and diabetic retinopathy.

## Methods

### Mice

C57BL/6, BALB/c, CB6F1/J (F1 progeny of C57Bl/6 and BALB/c), and *CX*_*3*_*CR1*^*YFP−CreER/YFP−CreER*^ [stock No. 021160] were purchased from Jackson Laboratories (Bar Harbor, Maine). The Cre reporter mouse line, *Rosa-lsl-tdTomato* (*R26*^*RFP*^), was kindly provided by the M.D. Gunn Lab (Duke University); mice were originally purchased from Jackson Laboratory [stock No. 007914]. The β-actin GFP transgenic mice were generated as previously described^54^ and were kindly provided by the G. H. Kelsoe Lab (Duke University). *CD11c-DTR/GFP* mice were kindly provided by the S.N. Abraham Lab (Duke University); mice were originally purchased from Jackson Laboratory [stock No. 004509]. All mice are housed at a barrier-free and specific-pathogen-free facility at Duke University School of Medicine (Durham, NC). All procedures were approved by the Institutional Animal Care and Use Committee at Duke University, and the procedures were carried out in accordance with the approved guidelines.

### RPE65 PCR

The PCR method to identify Methionine (Met) or Leucine (Leu) allele variations for the gene RPE65 was previously described^34^. We preformed the PCR on three different mouse strains: C57Bl/6J, BALB/c, and CB6F1/J (Fig. S6a) and found them to be Met/Met, Leu/Leu, and Met/Leu, respectively. The *CX*_*3*_*CR1*^*CreER−YFP/CreER−YFP*^ and *R26*^*RFP*^ strains were also confirmed to be RPE65 Met/Met (data not shown).

### Light challenge

Light damage is induced with energy/cost-efficient white LED lamps, as previously described^55^,^56^. These generate 90% less heat than current fluorescent lamps and emit short-wavelengths overlapping with rhodopsin absorption. After 8 hours of dark-adaption, mouse eyes were dilated with atropine, 1% solution (Bausch & Lomb, Tamp, FL) and phenylephrine hydrochloride, 10% solution (Paragon BioTeck, INC., Portland, OR). Mice were then placed in a ventilated reflective container that contained available food and hydrogel. A cool white-light LED light source (Fancierstudio, San Francisco, CA) was placed above the container and the lux output was adjusted to 60 k using a luxometer. After 8 hrs of light challenge, the mice were returned to normal mouse housing facility lighting.

C57Bl/6 mice are known to be resistant to light injury due to a Leu to Met mutation in the REP65 gene^34^. Our use of 60 k lux for 8 hrs is required for injury of our transgenic mice since they are on a C57Bl/6 (Met/Met) background (Fig. 5b). Lower intensity exposures (40 k lux/4hrs) in C57Bl/6 mice do not result in retina injury or subretina transmigration of Iba-1^+^ cells (Fig. S6b,c). In contrast, mice that contain at least on copy of the Leu RPE65 allele are susceptible to light injury at lower intensity exposures, as shown in Fig. S6b in BALB/c (Leu/Leu) and CB6F1/J (Leu/Met) mice (Fig. S6b,c).

### Labeling of intravascular CD45 cells

Mice were anesthetized by i.p. injections of ketamine/xylazine (120 and 20 mg/kg, respectively). Once anesthetized mice were injected retroorbitally with 3.0 ug of biotin-labeled anti-CD45 (Biolegend, San Diego, CA). Mice were euthanized 5 minutes post injection to ensure labeling of blood cells.

### Retina and blood harvest

Blood was collected from freshly euthanized mice via cardiac puncture. Cells were then treated with red blood cell lysis buffer (Sigma-Aldrich, St. Louis, MO) and thoroughly washed before immunostaining for flow cytometry. Eyes were gently enucleated. Using a dissection microscope, the cornea and lens were removed, exposing the retina. The retina was gently separated from the RPE using forceps and the optic nerve was severed at the back of the eye such that the retina could be isolated for digestion.

### Collagenase digestion

Single retinas were digested in 1.5 mg/ml collagenase A and 0.4 mg/ml DNase I (Roche, Indianapolis, IN) for 1 hour at 37 °C with agitation. Cells passed through a 70 μm filter, producing a single cell suspension.

### Flow cytometry immunolabeling

Single cell suspensions of retina or lysed blood were transferred into PBS for staining with Aqua Live/Dead viability dye (Thermo Fisher Scientific, Waltham, MA) for 30 minutes then washed. Cells were incubated in a blocking solution containing 5% normal mouse serum, 5% normal rat serum, and 1% Fc Block (eBiosciences, San Diego, CA) and subsequently stained with a combination of fluorophore-conjugated primary antibodies against CD45, Ly6C, Ly6G, CD64, CD11c, CD11b (Biolegend), F4/80 (eBiosciences), I-A/I-E (BD Biosciences, San Jose, CA) and CCR2 (R&D Systems, Minneapolis, MN). Samples were stained at room temperature for 20 minutes; then a fluorophore-conjugated streptavidin (Biolegend) was added to each sample and staining continued for an additional 5 minutes. After completion of staining, cells were washed and fixed with 0.4% paraformaldehyde in PBS. Data was acquired with BD Fortessa flow cytometer using BD FACSDiva software (BD Biosciences). Raw flow cytometry data was analyzed using FlowJo software (FlowJo LLC, Ashland, OR)

### Histology and immunolabeling

After removal of the cornea and lens, the eyecup (choroid and neuroretina) were fixed in 2% paraformaldehyde in PBS for 3 hrs at room temperature. Tissues were successively washed with PBS, 15% sucrose, and then 30% sucrose before embedding in OCT and frozen. Frozen sections were cut at 40 um for immunostaining with anti-GFP/YFP (Invitrogen) and DAPI. Z-stack images were collected using confocal microscopy (Leica) and image analysis preformed on Imaris (Bitplane, Zurich, Switzerland).

### Tamoxifen injections

Tamoxifen (Sigma-Aldrich) was dissolved in corn oil to a stock concentration of 20 mg/ml. 75 mg/kg of tamoxifen was i.p. injected twice with one day in between injections. Mice were approximately 5–6 weeks of age when given tamoxifen.

### Diphtheria toxin (DT) injections

A dose of 0.5 ug of DT (Sigma-Aldrich) was administered i.p. two days prior to harvest.

### Bone marrow chimeras

Chimeras were generated as previously described^57^. Recipient mice were lethally irradiated (1050 cGy). One cohort received whole-body exposure and another cohort was anesthetized then individually irradiated with lead shielding protecting their head. β-actin GFP transgenic donor mice were euthanatized and femurs and tibia collected. Distal and proximal ends of the bone were removed and the marrow flushed out with fresh RPMI medium. Single cell suspensions were thoroughly washed and resuspended in sterile HBSS. Immediately after irradiation, recipient mice received 5–10 million total GFP^+^ bone marrow cells in 200 μL via tail vein injection. Antibiotics were given in water to recipient mice.

### Statistics

FlowJo was used to generate the mean fluorescence intensities (MFI), standard deviations (s.d), and cell counts for each population of interest. Statistical significance of variations between populations was determined by t-test using Prism (GraphPad Software, Inc., La Jolla, CA).

## Additional Information

**How to cite this article**: O’Koren, E. G. *et al.* Fate mapping reveals that microglia and recruited monocyte-derived macrophages are definitively distinguishable by phenotype in the retina. *Sci. Rep.* **6**, 20636; doi: 10.1038/srep20636 (2016).

## Supplementary Material

Supplementary Information
