# Supplementary material for: Fate mapping reveals that microglia and recruited monocyte-derived macrophages are definitively distinguishable by phenotype in the retina
Source: Sci Rep. 2016 Feb 9;6:20636. doi: 10.1038/srep20636 (PMC4746646; doi:10.1038/srep20636)
Supplement: Supplementary Information [file srep20636-s1.pdf]

Fate mapping reveals that microglia and recruited monocyte-derived macrophages are definitively distinguishable by phenotype in the retina

O'Koren EG[1], Mathew R[1], Saban DR[1,2]

1. Department of Ophthalmology, Duke University School of Medicine, Durham, NC, USA

2. Department of Immunology, Duke University School of Medicine, Durham, NC, USA

SUPPLEMENTAL FIGURES

SUPPLEMENT FIGURE LEGENDS

Figure S1: MFI analysis of CD45, F4/80, CD11c and I-A/I-E expression on microglia vs. recruited mo-MFs in retina following whole-body irradiation/bone marrow transplantation. Mice were subjected to whole-body Rad/GFP-BMT; retinas were harvested 3 months later and MFI (mean ± s.d.) of each individual sample for the indicated markers are shown. Significant differences were determined within samples by an unpaired t test (****p < 0.0001). Data is representative of two independent experiments.

Figure S2: Analysis of myeloid cells in shielded Rad/GFP-BMT mice. (a) Complete chimerism cannot be achieved in the lead-shielded Rad/GFP-BMT setting. C57BL/6 mice underwent whole body or shielded Rad/GFP-BMT and FACS analysis was performed 3 months later. Cells were pre-gated on live $CD45^+$ $CD11b^+$ singlets. Unlike in whole-body Rad/GFP-BMT mice wherein IV-$CD45^+$ events were largely GFP^+, the shielded cohort possessed IV-$CD45^+$ events that were GFP^+ and GFP^- (GFP^- denoted by *). **(b)** MFI analysis of individual samples from shielded Rad/GFP-BMT hosts, which compares GFP^- microglia from uninjured retinas to GFP^+ mo-MFs from light-injured retinas. MFI (mean ± s.d.) of each individual sample for the indicated markers are shown. Significant differences were determined across samples by an unpaired t test (**p < 0.01, ****p < 0.0001).

Figure S3: Analysis in $CX_3CR1^{YFP\text{-}CreER/wt}$:$R26^{RFP}$ mice of myeloid cells during light injury. (a) Enumeration of microglia and mo-MFs in light injured-retinas. Mice were tamoxifen pulsed and 3 months later were subjected to light challenge (or not). Retinas were harvested 5 days later and prepared for flow cytometric enumeration. Data shows

the enumeration of total myeloid cells (YFP^+); microglia (YFP^+RFP^+); and recruited mo-MFs (YFP^+RFP^-) in uninjured and injured retinas. Data is a combination of 2 independent experiments (uninjured, n=3 individual samples; injured, n=7 individual samples). Significant differences were determined by an unpaired t test (**$p < 0.01$, *** $p < 0.001$). **(b, c)** Phenotypic comparison of extravasated $Ly6C^+$ Mo (I.), mo-MFs (II.) and microglia (III.). Mice were tamoxifen pulsed and 3 months later were subjected to light challenge (or not). Retinas were harvested 5 days later for flow cytometric analysis. Cells were pre-gated on live $CD45^+$ $CD11b^+$ IV-$CD45^-$ singlets. **(d)** Phenotypic analysis of microglia compared to mo-MFs following light injury in $CX_3CR1^{YFP\text{-}CreER/wt}$:$R26^{RFP}$ mice. Three months following tamoxifen pulsing, all mice were subjected to light challenge for subsequent flow cytometric analysis. MFI (mean ± s.d.) of each individual sample for the indicated markers are shown. Significant differences were determined within samples by an unpaired t test (****$p < 0.0001$). Data are representative of 2 independent experiments.

Figure S4: The $CD45^{lo}$ $CD11c^{lo}$ $F4/80^{lo}$ I-A/I-E^- phenotype is conserved for microglia both in steady state and during light injury. (a, b) Gating scheme and phenotypic analyses for normal retinal microglia, light-injured microglia, and recruited mo-MFs. Three months following tamoxifen pulsing, $CX_3CR1^{YFP\text{-}CreER/wt}$:$R26^{RFP}$ mice were subjected to light challenge (or not) and harvested 5 days later for subsequent flow cytometric analysis. Fluorescence minus one analysis using isotype controls are shown.

Figure S5: Analysis of both GFP^- and GFP^+ myeloid fractions in normal retina of *CD11c-DTR/GFP* mice. (a, b) Identification and enumeration of extravascular GFP^+ myeloid cells in the normal retina. Tissues were harvested from *CD11c-DTR/GFP* or control C57BL/6 mice. Two days prior to harvest, the former were treated with an i.p.

injection of 0.5 μg diphtheria toxin (DT). **(c)** Comprehensive phenotypic analysis reveals similarities between GFP^+ and GFP^- myeloid cells in the retina, namely $CD45^{lo}$ $CD11c^{lo}$ $F4/80^{lo}$ $I\text{-}A/I\text{-}E^-$. Data is representative of 2 independent experiments; each experiment has n=4-6 individual samples/group.

Figure S6: Characterization of LED light injury in different mouse strains. (a) PCR products of Leucine (Leu) or Methionine (Met) allele variants of the RPE65 gene across three mouse strains: BALB/c, C57Bl/6, and CB6F1/J. **(b)** Three mouse strains were subjected to light challenge (40k lux for 4 hr) and retinas were harvested 5 days later for retinal cross sections (outer nuclear layer = ONL; inner nuclear layer = INL; ganglion cell layer = GCL). After challenge, ONL thinning is visible in CB6F1/J and BALB/c mice, however there is no visible ONL thinning in C57Bl/6 mice. **(c)** Sections were stained with Iba-1 to visualize microglia and mo-MF recruits. Consistent with the presence of ONL thinning, CB6F1/J mice displayed migration of myeloid cells to the outer retina. **(d, e)** CB6F1/J and C57Bl/6 mice were subjected to light challenge (or not) and retinas were harvested 5 days later for flow cytometry. Data show gating using CD45 and CD11c to identify high and low expression cell populations (d), and subsequent analysis of I-A/I-E and F4/80 expression in each gated population. Cells were pre-gated on live $CD45^+$ $CD11b^+$ singlets. Data for this figure is representative of one experiment with an n=2 individual samples for each mouse strain.

Fig S1

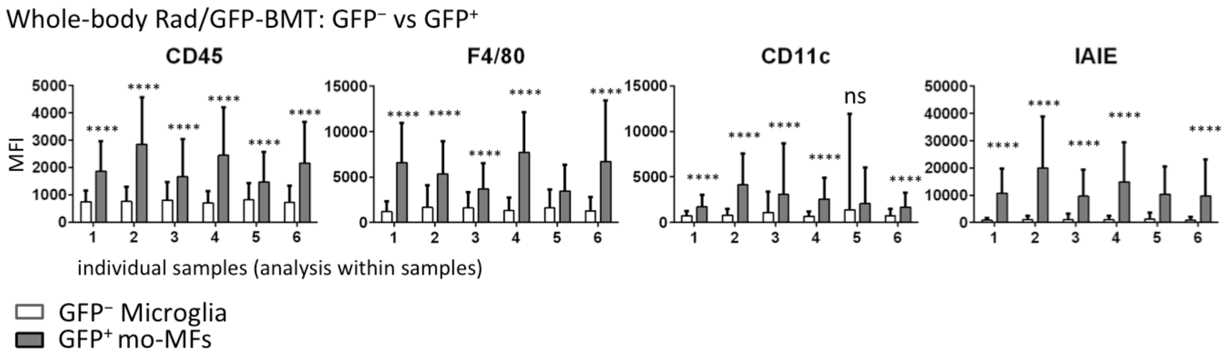

Whole-body Rad/GFP-BMT: GFP⁻ vs GFP⁺
CD45
F4/80
CD11c
IAIE
MFI
individual samples (analysis within samples)
ns
GFP⁻ Microglia
GFP⁺ mo-MFs

Fig S2

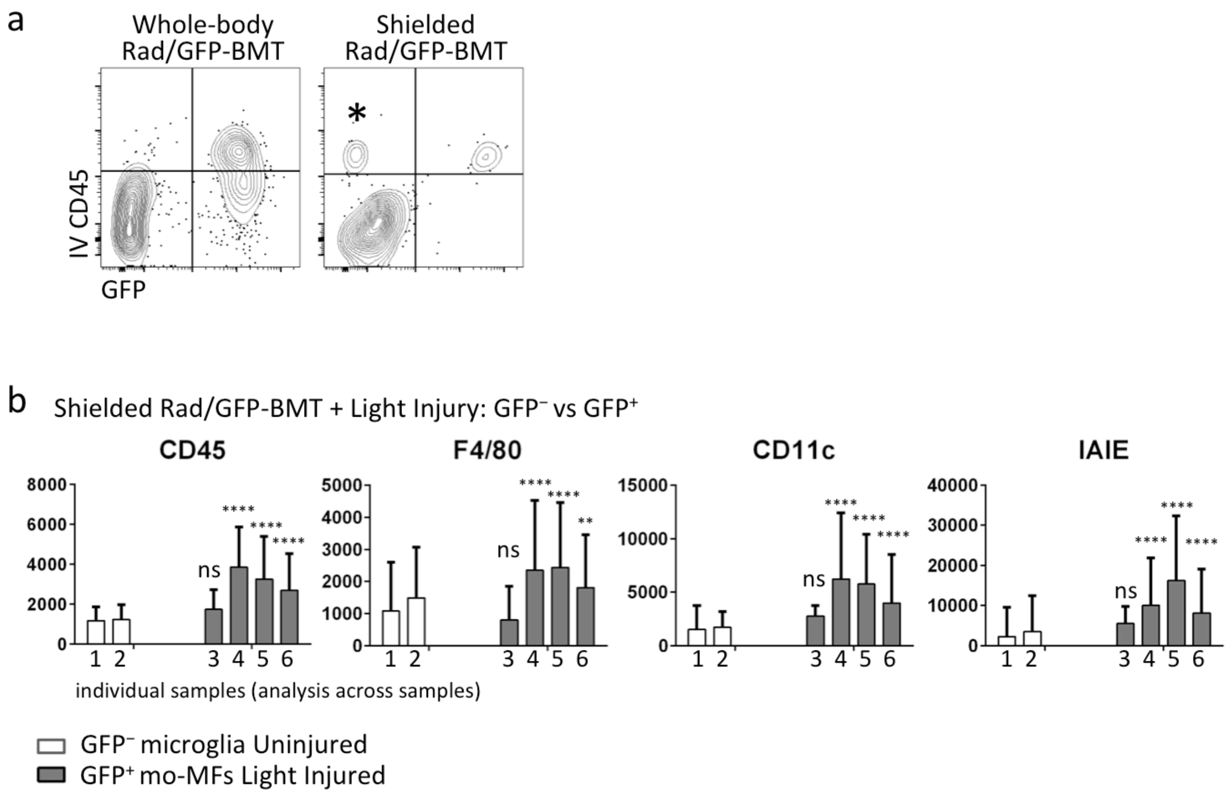

a
Whole-body Rad/GFP-BMT
Shielded Rad/GFP-BMT
*
IV CD45
GFP
b
Shielded Rad/GFP-BMT + Light Injury: GFP⁻ vs GFP⁺
CD45
F4/80
CD11c
IAIE
ns
****
**
1 2 3 4 5 6
individual samples (analysis across samples)
GFP⁻ microglia Uninjured
GFP⁺ mo-MFs Light Injured

Fig S3

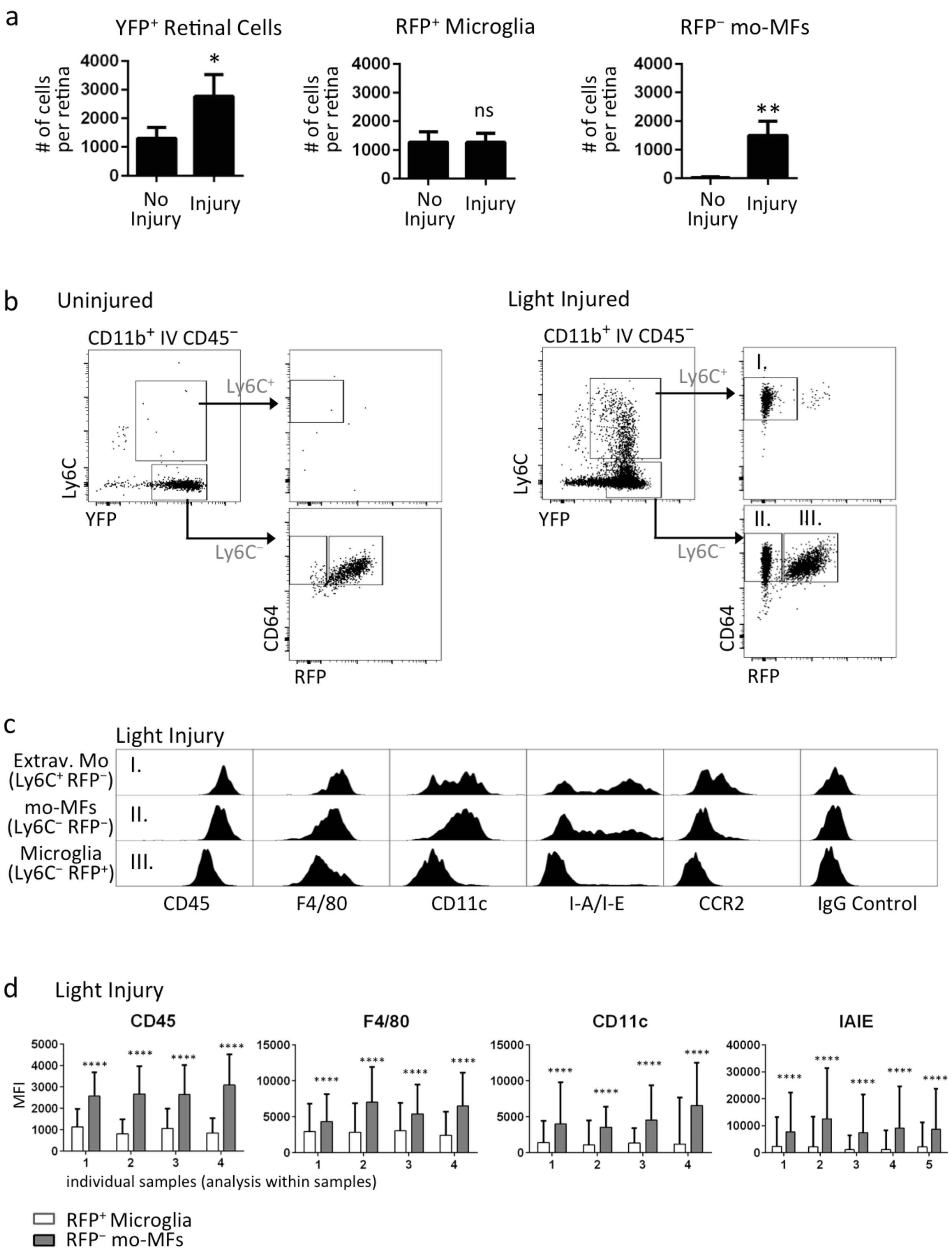
a
YFP⁺ Retinal Cells
RFP⁺ Microglia
RFP⁻ mo-MFs
# of cells per retina
4000
3000
2000
1000
0
*
ns
**
No Injury
Injury
b
Uninjured
Light Injured
CD11b⁺ IV CD45⁻
Ly6C⁺
Ly6C⁻
Ly6C
YFP
CD64
RFP
I.
II.
III.
c
Light Injury
Extrav. Mo (Ly6C⁺ RFP⁻)
mo-MFs (Ly6C⁻ RFP⁻)
Microglia (Ly6C⁻ RFP⁺)
CD45
F4/80
CD11c
I-A/I-E
CCR2
IgG Control
d
Light Injury
CD45
F4/80
CD11c
IAIE
MFI
****
individual samples (analysis within samples)
RFP⁺ Microglia
RFP⁻ mo-MFs

Fig S4

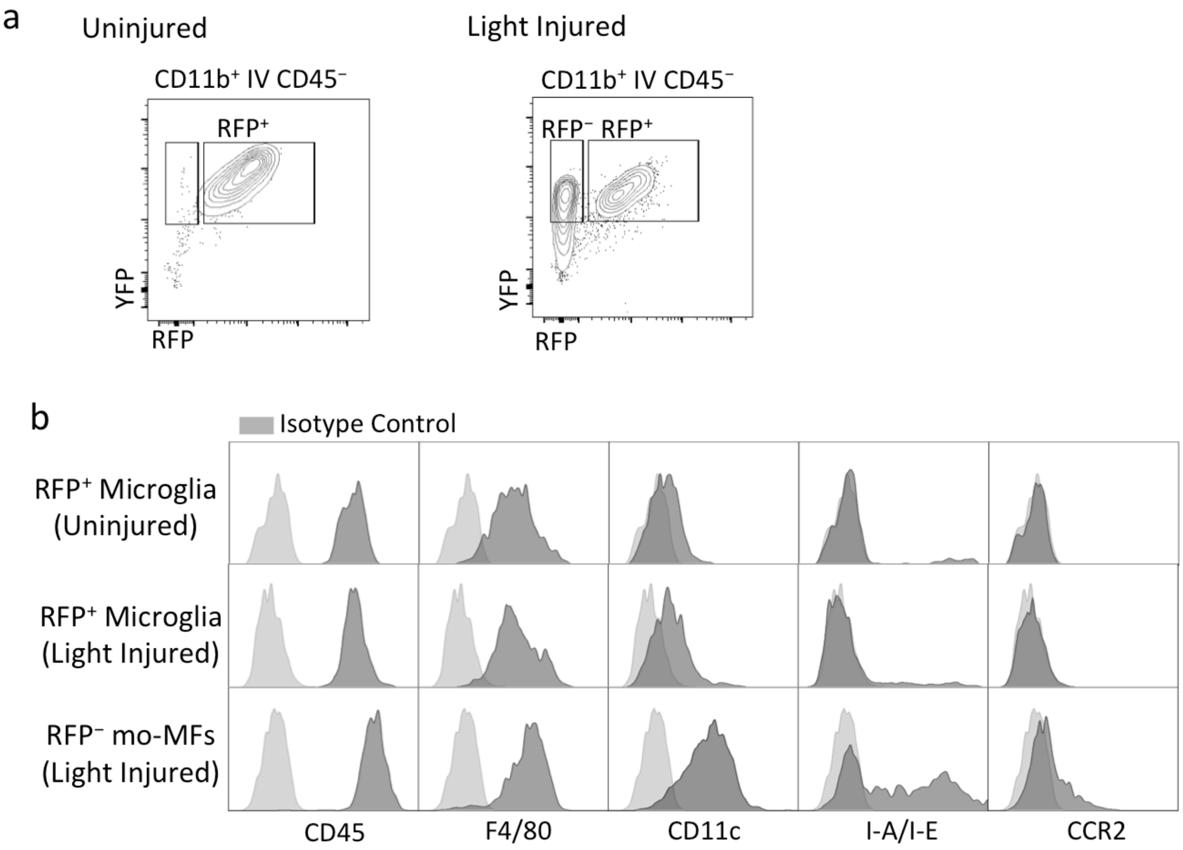
a
Uninjured
Light Injured
CD11b+ IV CD45−
RFP+
RFP−
YFP
RFP
b
Isotype Control
RFP+ Microglia (Uninjured)
RFP+ Microglia (Light Injured)
RFP− mo-MFs (Light Injured)
CD45
F4/80
CD11c
I-A/I-E
CCR2

Fig S5

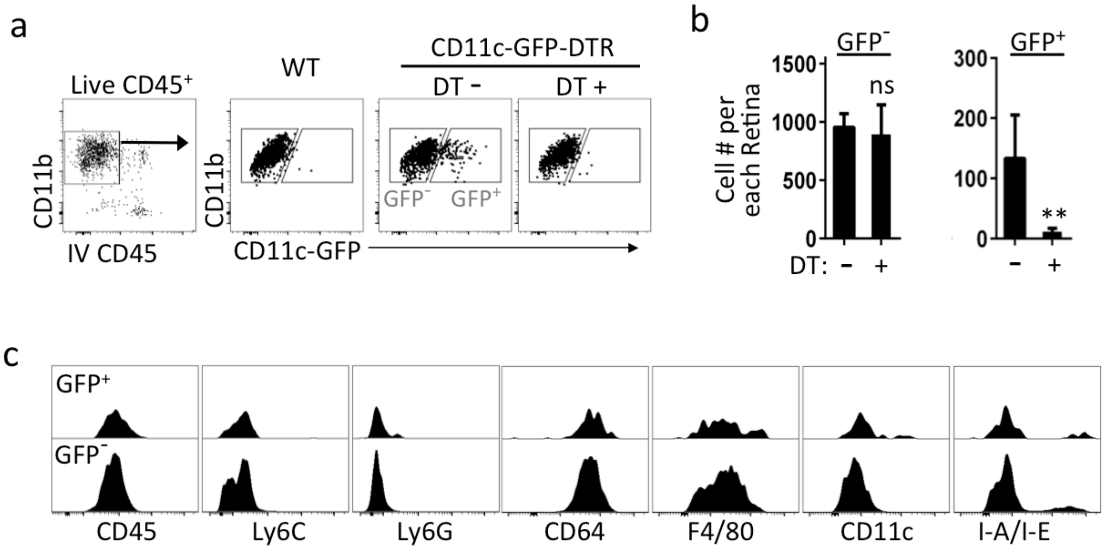
a
Live CD45+
CD11b
IV CD45
WT
CD11b
CD11c-GFP
CD11c-GFP-DTR
DT -
DT +
GFP-
GFP+
b
GFP-
GFP+
Cell # per each Retina
1500
1000
500
0
300
200
100
ns
**
DT:
-
+
c
GFP+
GFP-
CD45
Ly6C
Ly6G
CD64
F4/80
CD11c
I-A/I-E

Fig S6

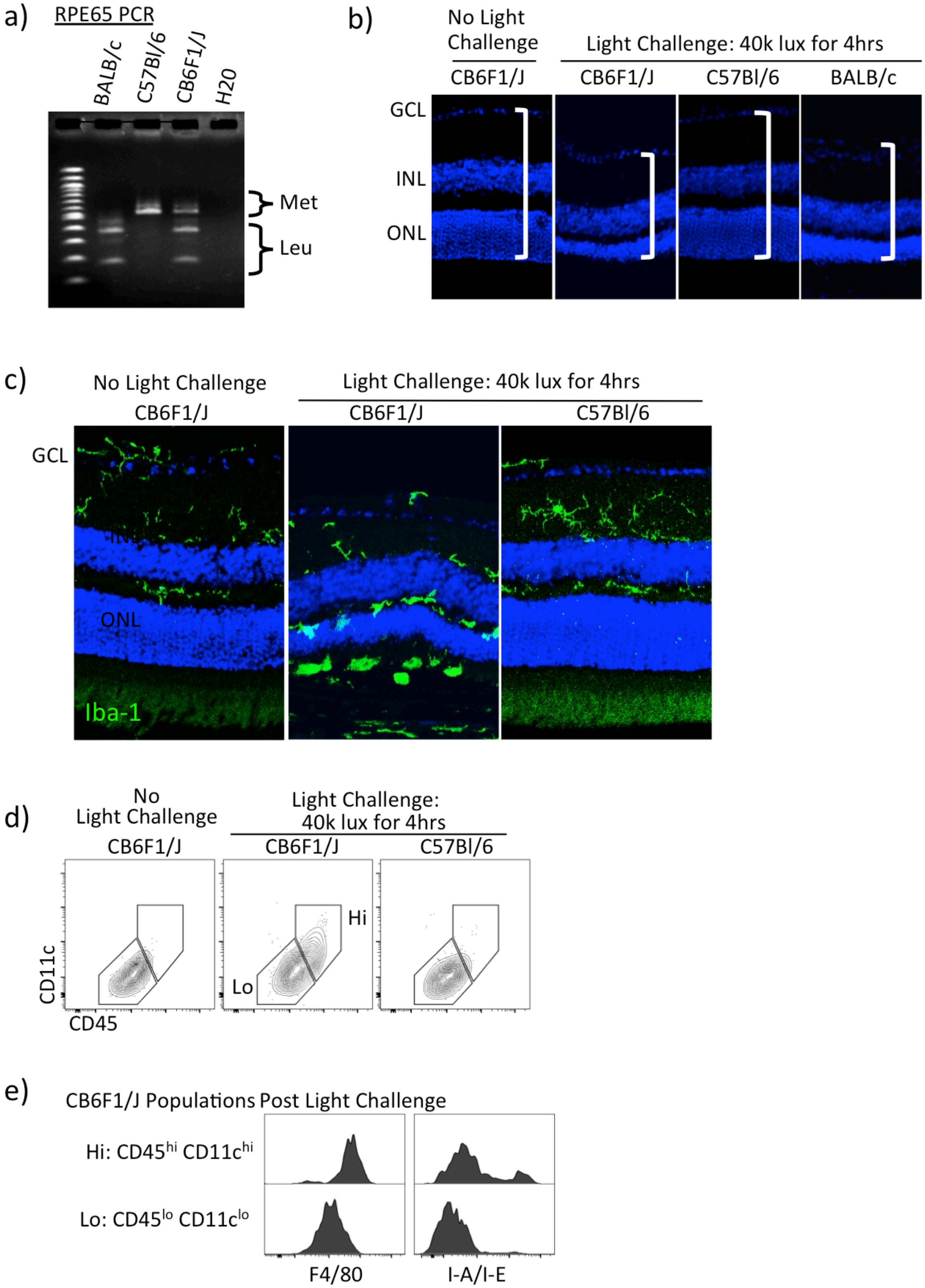
a)
RPE65 PCR
BALB/c
C57Bl/6
CB6F1/J
H20
Met
Leu
b)
No Light Challenge
Light Challenge: 40k lux for 4hrs
CB6F1/J
CB6F1/J
C57Bl/6
BALB/c
GCL
INL
ONL
c)
No Light Challenge
Light Challenge: 40k lux for 4hrs
CB6F1/J
CB6F1/J
C57Bl/6
GCL
INL
ONL
Iba-1
d)
No Light Challenge
Light Challenge: 40k lux for 4hrs
CB6F1/J
CB6F1/J
C57Bl/6
Hi
Lo
CD11c
CD45
e)
CB6F1/J Populations Post Light Challenge
Hi: CD45hi CD11chi
Lo: CD45lo CD11clo
F4/80
I-A/I-E
